# Integrating Susceptibility into Environmental Policy: An Analysis of the National Ambient Air Quality Standard for Lead

**DOI:** 10.3390/ijerph9041077

**Published:** 2012-03-27

**Authors:** Ramya Chari, Thomas A. Burke, Ronald H. White, Mary A. Fox

**Affiliations:** 1 RAND Corporation, 1200 South Hayes Street, Arlington, VA 22202, USA; 2 Department of Health Policy and Management, Johns Hopkins Bloomberg School of Public Health, 624 N. Broadway, Baltimore, MD 21205, USA; Email: tburke@jhsph.edu (T.A.B.); mfox@jhsph.edu (M.A.F.); 3 RHWhite Consulting, 12900 Tourmaline Terrace, Silver Spring, MD 20904, USA; Email: ronaldhwhite@comcast.net

**Keywords:** cumulative risk assessment, neurocognitive functioning, lead, nonchemical stressors, air standards, policy analysis

## Abstract

Susceptibility to chemical toxins has not been adequately addressed in risk assessment methodologies. As a result, environmental policies may fail to meet their fundamental goal of protecting the public from harm. This study examines how characterization of risk may change when susceptibility is explicitly considered in policy development; in particular we examine the process used by the U.S. Environmental Protection Agency (EPA) to set a National Ambient Air Quality Standard (NAAQS) for lead. To determine a NAAQS, EPA estimated air lead-related decreases in child neurocognitive function through a combination of multiple data elements including concentration-response (CR) functions. In this article, we present alternative scenarios for determining a lead NAAQS using CR functions developed in populations more susceptible to lead toxicity due to socioeconomic disadvantage. The use of CR functions developed in susceptible groups resulted in cognitive decrements greater than original EPA estimates. EPA’s analysis suggested that a standard level of 0.15 µg/m^3^ would fulfill decision criteria, but by incorporating susceptibility we found that options for the standard could reasonably be extended to lower levels. The use of data developed in susceptible populations would result in the selection of a more protective NAAQS under the same decision framework applied by EPA. Results are used to frame discussion regarding why cumulative risk assessment methodologies are needed to help inform policy development.

## 1. Introduction

To protect human health environmental chemical policies require accurate characterizations of both the exposure and the exposed. Individuals will be vulnerable to harm if they suffer from large chemical burdens but also if they exhibit increased susceptibility to toxic effects. Susceptibility to chemical toxins has not been adequately addressed in current risk assessment methodologies [1,2,3]. As a result, environmental policies may be failing to meet their fundamental goal of protecting the public from undue harm.

The U.S. Environmental Protection Agency (EPA) developed cumulative risk assessments (CRAs) as a means to address complexity in public health issues. Ideally CRAs would evaluate combined risks from aggregate exposures to multiple stressors–both chemical and nonchemical. Although susceptibility is an integral aspect of CRA [4], thus far the bulk of assessments have concentrated on risks from chemical mixtures [5]. In practice we often lack data regarding which populations are most susceptible and why they are more greatly affected. When data is sparse, the use of conservative assumptions is believed to result in policies that are protective of the most vulnerable groups. However, our limited knowledge about the extent and impact of susceptibility in the population undermines these assumptions.

The National Research Council (NRC) recently called for improving CRAs so that results would be more relevant to the needs and concerns of decision makers and affected communities [6]. One consequence of neglecting differential susceptibility in policy development might be the continuation of environmental health disparities. Individuals facing greater social and economic hardships tend to shoulder a greater exposure burden [7]. Due to the higher prevalence of potential chemical effect modifiers such as high stress levels, nutritional deficiencies, or co-morbid conditions, disadvantaged groups may also exhibit greater susceptibility to chemical toxicity [8,9,10].

To examine how consideration of susceptibility may affect policy decisions we examine the analytical process used by EPA to set a National Ambient Air Quality Standard (NAAQS) for lead (73 FR 66964) [11]. Lead is an ideal candidate for illustrating complexities surrounding incorporation of susceptibility into policy. The metal is differentially associated with socioeconomic status (SES), travels across multiple media and exposure pathways, accumulates within the body, does not exhibit a threshold, and induces dysfunction across numerous physiological systems [12]. The 2008 revised NAAQS was developed using an “air-related IQ loss framework” through which EPA evaluated risk assessment, experimental, and epidemiological data to quantify the relationship between air lead and neurocognitive deficits. Crucial to the overall assessment of risk is the choice of concentration-response (CR) function: an estimate relating changes in lead level to changes in health outcome. Specifically, EPA reviewed the epidemiological literature for CR functions assessing the relationship between blood lead and intelligence quotient (IQ) in children.

The CR functions evaluated by EPA represent the main effects of blood lead on IQ while controlling for other risk factors. Implicit in these estimates is the assumption that the relationship between lead and IQ does not vary across the levels of another factor. As numerous studies indicate however, this assumption may not hold true. Socioeconomic position has been shown to influence the extent of neurocognitive decline in a number of prospective [13,14,15,16] and cross-sectional studies [17,18,19]. Children in lower socioeconomic groups tend to exhibit greater loss of function than their more advantaged counterparts.

By defining the sensitive population of concern to be a highly exposed subset of children, one could argue that EPA did integrate susceptibility into their decision analysis. However to address concerns related to issues of environmental justice and equity, a focus on “policy-relevant” susceptibilities is required. By “policy-relevant” we refer to modifiable factors that can help explain persistent socioeconomic health disparities.

In this article, we present alternative scenarios for developing a lead NAAQS using CR functions developed in populations more highly susceptible to lead toxicity. Our objective is to examine how characterization of risk may change when susceptibility is explicitly and quantitatively considered in policy development. We focus on SES as a susceptibility factor because of its relevance for public health policy and prior evidence supporting its role as an effect modifier of lead toxicity.

Ultimately the most important question is whether the lead NAAQS protects human health with an “adequate margin of safety” as mandated under section 109 of the Clean Air Act (42 U.S.C. 7409) [20]. We contend that any analysis that does not include explicit consideration of susceptibility will be unable to provide a sufficient answer. The results of our analysis will illuminate why CRA is needed to inform policy development and will provide valuable lessons for evaluating cumulative risks within a policy decision framework.

## 2. Methods

### 2.1. EPA Analytical Approach

To assess the adequacy of the original 1978 lead NAAQS of 1.5 µg/m^3^, EPA undertook a lengthy and thorough review process, synthesizing over 6,000 scientific articles, soliciting expert and public comments, and performing a risk assessment (73 FR 66964) [11,21,22,23]. The standard itself consisted of four parts: an indicator, averaging time, form, and level. The 2008 revision set the lead NAAQS at a level of 0.15 µg/m^3^ with a rolling three-month averaging time and a maximum not-to-be-exceeded form, evaluated over a three-year period. The final standard arose from three key considerations encompassed in EPA’s decision framework: (1) choice of analysis parameters; (2) identification of the target population; and (3) determination of an acceptable risk level.

*Analysis Parameters*: The analysis required selection of: (1) CR functions; (2) air-to-blood lead ratios; and (3) potential standard levels. Firstly, to select CR functions EPA reviewed the scientific literature focusing on epidemiological studies that examined the effect of blood lead on IQ in children. After culling the dataset to four studies, the median CR function was chosen (−1.75 IQ points/µg/dL blood lead) to ward against undue influence from a single estimate (73 FR 67003) [11]. Secondly, based on available evidence [22,24], EPA evaluated a range of air-to-blood lead ratios developed in children (1:5, 1:7, 1:10). Ratios are presented as 1:x, with 1 representing air lead (µg/m^3^) and x representing blood lead (µg/dL). Finally, a range of potential lead standards were considered from 0.02 to 0.5 µg/m^3^.

*Target Population*: The EPA framework “provides estimates for that subset of children likely to be exposed to the level of the standard, which is generally expected to be the subpopulation of children living near sources who are likely to be most highly exposed…the framework does not provide estimates of the mean air-related IQ loss for all U.S. children” (73 FR 67005) [11].

*Acceptable Risk*: As EPA stated, “the Administrator concludes that an air-related IQ loss of 2 points should be used in conjunction with the evidence-based framework in selecting an appropriate level for the standard” (73 FR 67005) [11]. This decision was informed by the U.S. EPA Clean Air Scientific Advisory Committee which cautioned against a population mean IQ loss greater than 1–2 points in all but a small percentile of the population [25].

#### 2.1.1. Determining the Lead NAAQS

Table 1 displays the results of EPA’s analysis of expected target population mean IQ loss based on different levels of the standard, CR function, and air-to-blood lead ratio. IQ loss was calculated through the following equation:

β × A × B = IQ Loss   (1) 

where β is the slope of change in IQ associated with a 1 µg/dL increase in blood lead (β = −1.75 IQ points/µg/dL blood lead for all calculations), A is the potential standard level considered, and B is the air-to-blood lead ratio. 

**Table 1:** Expected mean IQ loss estimates for children exposed at the level of the standard.

Potential Level for Standard (µg/m^3^)	Air-to-Blood Lead Ratio*		
	1:5	1:7	1:10
0.50	4.4 (3.9–7.4)	>5 ^a^	>5 ^a^
0.40	3.5 (3.1–5.9)	4.9 (4.4–8.2)	^b^
0.30	2.6 (2.3–4.4)	3.7 (3.3–6.2)	5.3 (4.7–8.8)
0.25	2.2 (2.0–3.7)	3.1 (2.7–5.1)	4.4 (3.9–7.4)
0.20	1.8 (1.6–2.9)	2.5 (2.2–4.1)	3.5 (3.1–5.9)
0.15	1.3 (1.2–2.2)	**1.8 (1.6–3.1)**^c^	2.6 (2.3–4.4)
0.10	0.9 (0.8–1.5)	1.2 (1.1–2.1)	1.8 (1.6–2.9)
0.05	0.4 (0.4–0.7)	0.6 (0.5–1.0)	0.9 (0.8–1.5)
0.02	0.2 (0.2–0.3)	0.2 (0.2–0.4)	0.4 (0.3–0.6)

* Ranges are based on the lowest and highest concentration-response functions. ^a^ For these combinations of air-to-blood ratio and standard, the resulting estimate is too uncertain to state with precision; ^b^ Not reported; ^c^ Final standard level and associated mean IQ loss highlighted for emphasis; Reference: 73 FR 66964 [11].

As shown in Table 1, under an air-to-blood lead ratio of 1:7 a standard of 0.15 µg/m^3^ would result in a loss of <2 IQ points in the target population mean, meeting the acceptable risk criterion. We note that EPA undertook a careful evaluation of other aspects of the lead standard. Choice of averaging time and form was also directly relevant to the selection of a final standard, though we do not focus on these properties in our analysis. It is the standard level of 0.15 µg/m^3^ *in conjunction with* a rolling three-month averaging time and a maximum not-to-be-exceeded form that “would significantly reduce and limit for a high percentage of U.S. children the risk of experiencing an air-related IQ loss of (2 points)” (FR 73 67005) [11]. 

### 2.2. Incorporation of Susceptibility

While there were numerous considerations that were involved in the selection of a lead NAAQS, our analysis focused mainly on the choice of CR function. We conserved the standard levels EPA evaluated as well as child air-to-blood lead ratios.

To identify candidate CR functions we relied on a previously conducted systematic review of studies assessing SES as an effect modifier of lead toxicity for neurocognitive, renal, and cardiovascular (hypertension) outcomes [26]. CR functions for neurocognitive outcomes were included in the present analysis if their associated studies focused on children, measured lead in blood, assessed global aspects of cognitive function (Mental Development Index (MDI) or IQ), and reported separate estimates for each socioeconomic level.

#### 2.2.1. Data Analysis

We considered various methods for combining individual study CR functions, from simple median values to inverse variance weighting by susceptibility subgroup. Following EPA’s methods, we included a range of standard levels and air-to-blood lead ratios in our analysis. Expected outcomes (neurocognitive abilities in children) associated with different standard levels were determined according to the following equation:

β_j_ × A × B = y_j_   (2) 

where A is the potential standard level, β_j_ is the regression coefficient relating the expected change in cognitive ability associated with a 1 µg/dL increase in blood lead in susceptibility subgroup j, B is the child-specific air-to-blood lead ratio, and y_j_ is the resulting neurocognitive outcome in susceptibility subgroup j (e.g., loss of IQ or MDI points). 

## 3. Results

### 3.1. Description of Data

Table 2 presents descriptions of the CR functions considered for the analysis. Four neurocognitive studies met our inclusion criteria [16,27,28,29]. Three studies were prospective in design [16,28,29] and one was cross-sectional [27]. The three prospective studies actually represent data from two cohorts evaluated over time. Dietrich *et al*. [28] focused on a cohort of Cincinnati-based infants assessed at six months. (We initially identified two studies by Dietrich *et al*. [14,28]; both focused on infants assessed at six months–however the form of lead differed between studies (untransformed [14] *versus* natural log transformation [28]). We chose to include the function involving a log transformation of blood lead because of the large research base supporting the nonlinear relationship between lead and neurocognitive function [21].) Both McMichael *et al*. [29] and Tong *et al*. [16] analyzed data from children living in the areas surrounding a lead smelter in Port Pirie, Australia. 

**Table 2:** Concentration-response functions considered in the analysis.

β (SE or 95th CI if reported) ^a^	Susceptibility	Lead Form	Lead Measure	Overall Lead Mean (µg/dL)	Outcome	Age at Assessment	Reference
			
**Studies used by EPA to determine concentration-response function**							
−1.71	Overall	Linear	Concurrent	2.9 (0.8–4.9) ^b,c^	MDI (BSID)	24 months	Tellez-Rojo *et al*. [30]
−2.94	Overall	Linear	Concurrent	3.2 (0.9–7.4) ^b,c^	IQ (WISC-R)	4–10 years	Lanphear *et al*. [31] ^d^
−1.79	Overall	Linear	Concurrent	3.3 (0.5–8.4) ^b,c^	IQ (SBIS)	5 years	Canfield *et al*. [32]
−1.56	Overall	Linear	Neonatal	3.8 (1.0–9.3) ^b,c^	IQ (WISC-R)	10 years	Bellinger & Needleman [33]
**Studies with SES-subgroup specific effects included in the analysis**							
−4.70	Low SES	Ln	Neonatal	4.5 (2.9)	MDI (BSID)	6 months	Dietrich *et al*. [28]
−4.90 ^e^	Low SES	Ln	Average Postnatal	21.3	MDI (BSID)	2 years	McMichael *et al*. [29]
−4.57 ^e^	Middle SES						
−1.87 ^e^	High SES			(13.3–33.8) ^f^			
−9.60 (3.60)	Low SES	Ln	Lifetime Average	14.1 (1.2) ^b^	IQ (WISC-R)	11–13 years	Tong *et al*. [16]
−2.90 (3.40)	High SES						
−0.03	Manual	Linear	Concurrent	15.6 (4.1)	IQ (BAS)	2–6 years	Harvey *et al*. [27]

^a^ Estimates are interpreted as change in test score per 1 µg/dL increase in blood lead level; ^b^ Geometric mean; ^c^ Range (minimum-maximum); ^d^ The Lanphear *et al*. [31] pooled study included data from the cohorts analyzed by Canfield *et al*. [32] and Bellinger and Needleman [33] but for different ages; ^e^ Unadjusted estimates; ^f^ Range (25th to 75th percentile). Abbreviations: SES (socioeconomic status); MDI (Mental Development Index); BSID (Bayley Scales of Infant Development); IQ (intelligence quotient); SBIS (Stanford-Binet Intelligence Scale); WISC-R (Wechsler Intelligence Scale for Children-Revised); BAS (British Ability Scales).

The presence of the lead smelter may explain the higher population mean blood lead levels found in the Port Pirie *versus* Cincinnati cohort (Table 2). For comparison purposes, Table 2 also includes the four studies chosen by EPA to determine the CR function used in their analysis to select the lead standard [30,31,32,33].

**Figure 1:**
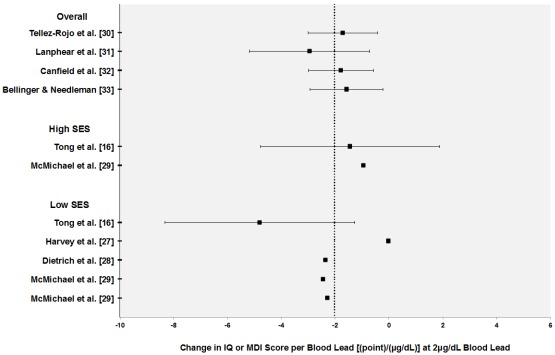
Concentration-response (CR) functions for neurocognitive outcomes in children (intelligence quotient and mental development index) grouped according to socioeconomic status. CR functions are calculated at 2 µg/dL blood lead level. Error bars represent 95% confidence intervals. Vertical dotted line represents mean CR estimates across studies.

Figure 1 displays individual CR functions grouped according to SES. To interpret the nonlinear models, we calculated slopes at a blood lead level of 2 µg/dL. Many of the neurocognitive studies did not report enough information to calculate variability estimates. Overall there do appear to be slight shifts in the distribution of functions based on susceptibility group. CR functions developed in low and high SES groups tend to fall below and above the mean respectively.

### 3.2. Selection of Parameters and Analysis Results

#### 3.2.1. Concentration-Response Functions

For susceptibility subgroups with multiple CR functions we determined both the median value and a pooled estimate using inverse variance weighting and random effects methods (Table 3). We were unable to calculate pooled estimates for all groups because of lack of reported variability estimates in the neurocognitive studies. For nonlinear models, the CR functions in Table 3 represent the effect of blood lead at 2 µg/dL. We chose this level as the most recent (2007–2008) NHANES data indicates that the mean lead levels in the United States for various age groupings tends to fall under 2 µg/dL. If studies reported low, medium, and high SES estimates, we only used high and low SES CR functions to simplify comparisons.

**Table 3:** Individual and combined concentration-response functions at 2 µg/dL blood lead level.

Reference	Susceptibility	CR Function (95% CI)	Median CR (95% CI)	Pooled CR (95% CI)
				
Tellez-Rojo *et al*. [30]	Overall	−1.71 (−3.00, −0.42)	−1.75 (−3.00, −0.51)	−1.82 (−2.52, −1.12)
Lanphear *et al*. [31]	Overall	−2.94 (−5.16, −0.71)		
Canfield *et al*. [32]	Overall	−1.79 (−3.00, −0.60)		
Bellinger & Needleman [33]	Overall	−1.56 (−2.90, −0.20)		
				
McMichael *et al*. [29]	High SES	−0.94	-1.19	NA
Tong *et al*. [16]	High SES	−1.45 (−4.78, 1.88)		
				
Dietrich *et al*. [28]	Low SES	−2.35	−2.40	NA
McMichael *et al*. [29]	Low SES	−2.45		
Tong *et al*. [16]	Low SES	−4.80 (−8.33, −1.27)		
Harvey *et al*. [27]	Low SES	−0.03		

Abbreviations: CR (concentration-response); SES (socioeconomic status); HS (high school).

#### 3.2.2. Ratios and Standard Levels

Acknowledging uncertainty in the estimates, EPA’s final analysis for the revision of the NAAQS consisted of a range of child-specific ratios with 1:5, 1:10, and 1:7 representing the lower and upper bounds, and central estimate respectively. We used these ratios in our analysis.

Based on a review of scientific evidence, expert recommendations, and public comments, EPA considered a range of levels for the new standard (0.02, 0.05, 0.10, 0.15, 0.20, 0.25, 0.30, 0.40, and 0.50 µg/m^3^) (73 FR 66996) [11]. EPA noted that many commentators recommended an upper level of 0.20 µg/m^3^ for the final standard. We retain the same range of levels for our analyses.

### 3.3. Analysis Results

Table 4 displays expected IQ loss associated with different levels of the air lead standard using a CR function developed in low SES groups (β = −2.40 points/µg/dL blood lead). Results are presented using median CR estimates to represent susceptibility subgroups with multiple functions available. (Similar results were found using pooled estimates.) Compared to the values in Table 1, at all potential standard levels we note a greater expected decrease in population mean IQ using these susceptibility-specific estimates. Under the 0.15 µg/m^3^ standard, the loss in IQ at the central air-to-blood lead ratio exceeds the 2-point acceptable risk level set by EPA (73 FR 67005) [11].

**Table 4:** Expected mean intelligence loss estimates for children at different air lead standard levels using concentration-response functions developed in low socioeconomic groups *.

Potential Standard (µg/m^3^)	Air-to-Blood Lead Ratio		
	1:5	1:7	1:10
0.02	0.2 (0.00, 0.5)	0.3 (0.00, 0.7)	0.5 (0.01, 0.1)
0.05	0.6 (0.01, 1.2)	0.8 (0.01, 1.7)	1.2 (0.02, 2.4)
0.10	1.2 (0.02, 2.4)	1.7 (0.02, 3.4)	2.4 (0.03, 4.8)
0.15	1.8 (0.02, 3.6)	**2.5 (0.03, 5.0)**	3.6 (0.1, 7.2)
0.20	2.4 (0.03, 4.8)	3.4 (0.04, 6.7)	4.8 (0.1, 9.6)
0.25	3.0 (0.04, 6.0)	4.2 (0.1, 8.4)	6.0 (0.1, 12.0)
0.30	3.6 (0.15, 7.2)	5.0 (0.1, 10.1)	7.2 (0.1, 14.4)
0.40	4.8 (0.16, 9.6)	6.7 (0.1, 13.4)	9.6 (0.1, 19.2)
0.50	6.0 (0.1, 12.0)	8.4 (0.1, 16.8)	12.0 (0.2, 24.0)

* Ranges are based on the lowest and highest concentration-response functions.

## 4. Discussion

### 4.1. Overview of Results

To determine the NAAQS for lead, EPA developed an evidence-based framework for estimating IQ loss from exposure to lead in air. Expected decreases in function were determined through an analysis and combination of multiple data elements. The process was informed by an important decision criterion–EPA would reject any standard that resulted in greater than a 2-point IQ loss in the population mean of children exposed at the level of the standard.

EPA defined children as the most sensitive population but did not consider additional susceptibilities that might modify the adverse effects of lead. The Agency’s original analysis used a CR function that resulted in an estimated mean IQ loss of 1.8 points at the 0.15 µg/m^3^ standard level. Our analysis showed that the use of CR functions developed in groups more susceptible to lead toxicity resulted in cognitive decrements that exceeded the acceptable risk level set by EPA (−2.5 IQ loss/µg/dL blood lead, Table 4). Retaining EPA’s decision criterion, a lower standard of 0.10 µg/m^3^ would be necessary to protect against a greater than 2-point IQ loss in the target population mean.

The potential for children of greater socioeconomic disadvantage to display greater susceptibility to lead is an important concern–especially as EPA’s IQ-loss framework specifically refers to the subset of children who would likely be exposed at the level of the standard (73 FR 67005) [11]. While we lack the data to accurately quantify or characterize this group, it is possible that children more likely to live near lead sources (and thus be exposed at the standard level) are also more likely to suffer economic disadvantage [34,35,36]. By using CR functions that do not account for the possibility of increased susceptibility in this group, EPA’s IQ-loss framework may not protect the target population to the desired extent.

#### 4.1.1. Analysis Limitations

Our analysis was a critical exercise aimed at evaluating how susceptibility could be explicitly incorporated into environmental policy in order to draw lessons for risk assessment. Our final estimates are subject to uncertainty primarily due to inadequacies in the database. We note some key differences between the CR functions used in our approach *versus* EPA’s analysis that affect the comparability of our estimates.

Given the evidence of nonlinearity between blood lead level and IQ loss (where a steeper slope occurs at lower lead concentrations), EPA selected CR functions developed in study populations with blood lead levels closest to the low levels currently experienced by children. Because of this nonlinear relationship, CR functions derived from populations with higher mean lead levels would actually underestimate the adverse effects of lead in children today. Unfortunately the neurocognitive studies we identified were all conducted in cohorts with mean lead levels much greater than current levels (~4 to 15 *versus* 2 µg/dL).

In addition, EPA’s analysis focused on concurrent measures of blood lead and neurocognitive function because of evidence supporting a strong association between the two parameters. However, of the studies we identified that explored socioeconomic differences in lead effects, only one used concurrent measures of blood lead; the rest used lifetime average or neonatal blood lead measures.

Finally, we note that at the present time, the epidemiological literature provides only suggestive evidence of SES as an effect modifier of lead neurotoxicity [26]. In Chari *et al*. [26], we identified forty studies that explored differential lead effects across SES groups; however only a small subset of these studies provided enough information to determine socioeconomic subgroup estimates. The four studies used in our analysis do not by themselves provide definitive evidence about the existence or magnitude of socioeconomic interactions with lead. It is also important to consider that different SES measures were used across the studies, potentially hindering comparability. Additionally, in the Cincinnati cohort, the authors found significant interactions between SES and lead in earlier but not later years indicating that effects may not persist or may be attenuated by undetermined circumstances. The effect of socioeconomic position on lead neurotoxicity is a complex phenomenon and our analysis presents a simplified assessment in order to draw larger policy implications.

### 4.2. Role for Cumulative Risk Assessment in Policy Development

As part of their methodology, EPA performed a traditional risk assessment to support the development of the lead NAAQS; however uncertainties and limitations prevented the Agency from relying on the final estimates. EPA noted that the multimedia and persistent nature of lead along with the potential for multiple exposure pathways made the risk assessment considerably more complex than assessments for other criteria pollutants that focused on inhalation exposures only (73 FR 66979) [11]. Regardless, risk assessment results were used to provide additional perspectives on expected IQ loss resulting from air-lead exposure. Four CR functions were derived from an international pooled analysis [31] to account for uncertainty in the exposure-outcome relationship at low blood lead concentrations [22]. Differential susceptibility was not considered in the risk assessment (beyond focusing on children as the sensitive population).

Though central to CRA, we should note that issues of susceptibility have been considered in traditional risk assessments. For noncancer endpoints, EPA estimates acceptable exposure levels or reference doses (RfDs)–the amount of daily exposure to chemical toxins unlikely to result in harm over a lifetime [37]. In the estimation of the RfD, 10-fold uncertainty factors are typically applied to account for interspecies extrapolation, human variation in sensitivity, lack of chronic data, as well as other data deficiencies. The use of a single 10-fold uncertainty factor for human variation in susceptibility has been questioned however, especially in light of more recent efforts to address cumulative exposures to multiple chemicals, inter-individual variability in health risk factors, and the clustering of multiple vulnerability factors within individuals or populations [1,2,3]. For cancer endpoints, slope factors represent the upper bound on the average population risk and EPA considers the use of upper bounds to be protective for susceptible populations [2]. However, risks can be underestimated if the slope factors are derived from studies on populations that have less susceptibility that average (e.g., healthy workers) or do not include exposures during susceptible life stages in the design.

Overall, risk assessment guidelines recommend incorporating information about susceptibility into all steps of the process [38,39,40,41]. EPA acknowledges however, that in practice we usually lack information about the range of susceptibility in a population. Furthermore, in its guidelines, the Agency tends to emphasize intrinsic susceptibility characteristics (fixed, inherent aspects of an individual) such as genetic polymorphisms or metabolism rates, and excludes consideration of acquired characteristics (variable, modifiable traits) such as psychological stress or SES [7]. With its emphasis on combined risks, CRA is well suited to address the broader acquired characteristics. Schwartz *et al*. [42] note that the focus on intrinsic susceptibility characteristics may arise from the fact that presently, these data are easier to collect and analyze. However upstream susceptibility factors, such as SES may in fact be the more important drivers of environmental health disparities. While both intrinsic and acquired susceptibility factors are important, the latter are especially valuable for public health purposes as these factors are amenable to intervention.

#### 4.2.1. Susceptibility and Decision-making

The legislative history of the Clean Air Act, Section 9, includes a discussion of those who primary ambient air quality standards should protect:

“…included among those persons whose health should be protected by the ambient standard are particularly sensitive citizens…who in the normal course of daily activity are exposed to the ambient environment…an ambient air quality standard, therefore, should be the maximum permissible ambient air level of an air pollution agent…which will protect the health of any group in the population.” (S. Rep. No. 91-1196, 91st Cong., 2d Sess. 10 (1970)) [43].

Based on the language of the Senate Committee Report accompanying the Clean Air Act, air standards should be considered “adequate” if they are stringent enough to protect especially sensitive groups. Consideration of susceptibility thus appears to be a legislative requirement for the development of the NAAQS.

However, even after the selection of a sensitive population such as children, dealing with variability in response to exposure remains a difficult task, made even more so when data are inadequate to characterize the extent of variability. In the absence of data, research indicates that individuals are “quite bad at making complex, unaided decisions” [44]. Without explicit and quantitative consideration of susceptibility, policy decisions may be less protective and less equitable [45]. Quantifying susceptibility will allow for more informed choices, better assessment of decision consequences, and increased accountability and transparency in policy development. A number of specific policy issues arose from our analysis that illustrate why better quantification of acquired susceptibility factors is needed to improve decision-making.

#### 4.2.2. Defining Acceptable Risks

The determination of an acceptable risk level is a value-laden policy judgment and represents one type of decision uncertainty [46,47]. This type of uncertainty arises when there is ambiguity regarding how to value social objectives. EPA was careful in describing its choice of a <2 point IQ loss to guide policy decisions stating that,

“EPA is not determining a specific quantitative public health policy goal in terms of an air-related IQ loss that is acceptable or unacceptable in the U.S. population per se, but instead is determining what magnitude of estimated air-related IQ loss should be used in conjunction with the specific…evidence-based framework being applied in this review, recognizing the uncertainties and limitations in this framework…the estimated air-related IQ loss resulting from the application of this evidence-based framework should not be viewed as a bright line estimate of expected IQ loss in the population that would or would not occur. Nonetheless, these results provide a useful guide…to use in making the basically qualitative public health policy judgment about the risk to public health that could reasonably be expected to result from exposure….” (FR 73 67000) [11].

**Figure 2:**
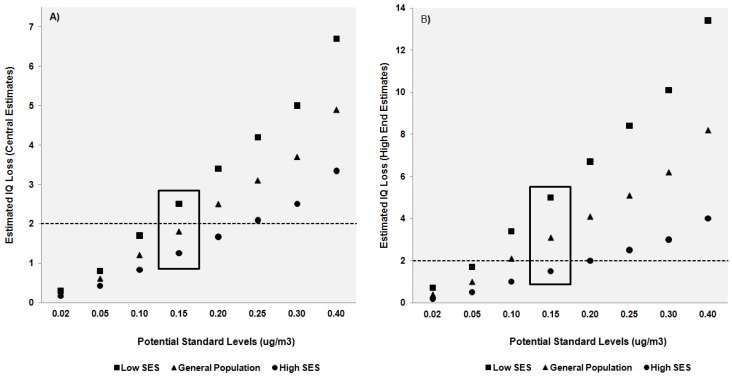
Comparison of IQ loss estimates derived from general population and susceptible groups for two scenarios: (**A**) central estimates derived from median concentration-response (CR) functions; and (**B**) high-end estimates generated from the largest available CR function in each population (see Table 3). Differences across population groups are grouped according to potential standard level. Dotted line at 2 IQ points represents the acceptable risk level defined by EPA. A box surrounds estimates associated with the final standard.

EPA’s decision to use a loss of 2 IQ points as a criterion was not made prior to carrying out the analysis. Instead the Agency concluded that this acceptable risk level was a fair judgment given the various decision elements, expert and public comments, and uncertainty in the estimates. However as noted by Hattis and Anderson [45], quantitative assessment of variability can lead to “richer and more accurate information available for decision-making.” Even if data are not sufficient to base a decision on, the process of gathering and assessing available information may help inform policy-decision frameworks. As an example, Figure 2 graphically illustrates the range of IQ loss estimates expected at each potential level of the standard comparing the results of EPA’s original analysis with estimates generated from CR functions developed in low- and high-socioeconomic groups. Figure 2a is a simple graphical depiction of data shown in Table 1 for IQ loss estimates associated with the central air-to-blood lead ratio. Figure 2b depicts “high-end” estimates generated from the largest CR functions available in each population group (see Table 3). In both figures, results using EPA’s analysis parameters are depicted with triangle symbols and fall in the middle of IQ loss estimates generated from our analysis using susceptibility specific data. At standard levels above 0.15 µg/m^3^ only estimates derived from high-SES groups (circle symbol) fall under or close to the acceptable risk level. At standards below a level of 0.10 µg/m^3^ loss estimates from all groups meet acceptability requirements. 

EPA’s analysis suggested that a level of 0.15 µg/m^3^ would fulfill decision criteria, but by incorporating susceptibility we see that standard considerations could reasonably be extended to 0.10 µg/m^3^. EPA considered its final judgments adequate due to a tendency to make conservative decisions across multiple decision elements. Even if an explicit accounting of susceptibility does not change final decisions, the analysis itself is important for increasing the transparency of a decision process filled with uncertain inputs. 

As Rodricks [47] notes, despite their importance, acceptable risk issues have received little public health evaluation. Lead is an especially difficult pollutant to assess because of its ubiquity and the lack of a threshold under which no adverse effects occur. Because of this reality, policymakers must make controversial choices regarding what level of risk, or loss of function is acceptable and to whom. Furthermore, unlike with carcinogens, these decisions must be made without any historical guidance. The NRC has recommended that EPA unify cancer and noncancer dose-response assessments which may result in a greater number of “noncancer” chemicals evaluated under a no-threshold model [6]. EPA has already noted the lack of accepted criteria within the public health community for the appropriate degree of protection afforded to neurocognitive effects in susceptible populations (FR 73 66997) [11]; if NRC recommendations come to pass, in the future we can expect to see less regulatory “bright lines” indicating safe exposure levels and more deliberation over acceptable risks. Susceptibility should be central to these conversations. Risk decisions are already difficult, controversial, and subject to criticism from stakeholders [7]. The determination and acceptability of an “acceptable” risk depends on the population to which it is applied. To be effective, relevant, and scientifically justifiable, environmental policies must account for susceptible populations.

#### 4.2.3. Prioritizing Decision Elements

Quantitative information on susceptibility can benefit decision-making by directly addressing issues of inequity and helping to target interventions [47]. Both of these issues require adequate identification of susceptible groups and characterization of expected effects within each group. Any “one-size-fits-all” standard will result in variable effects because of variability in both exposure and susceptibility across a population. The result will be continued disparities in adverse outcomes due to environmental exposure. Data on susceptibility would help to identify exactly which groups may not be sufficiently protected by the current lead NAAQS for example. Additional “downstream” interventions could then be targeted towards those populations. 

During policy development information about susceptibility can be used to inform the process by allowing comparisons across vulnerable subpopulations. The base of research currently best substantiates child neurological and adult cardiovascular functioning as most sensitive to lead at low blood levels. Lead bioaccumulates in the body, with bone serving as the largest storage component. Throughout life, lead is exchanged between blood and bone as well as blood and soft tissues [21]. Based on the fact that blood lead levels in adulthood are influenced by previous exposures, EPA gave precedence to children as the susceptible subpopulation and reasoned that the lead NAAQS should offer adequate protection against cardiovascular and renal effect in adults (73 FR 66975) [11]. While children and neurotoxicity are important targets for public health intervention, it is possible that other susceptibility groups and health outcomes warrant explicit consideration within a decision framework.

### 4.3. Data Needs for Cumulative Risk Assessment

As mentioned previously, CRA is an ideal framework for integrating acquired susceptibility factors into research and policy. Unlike intrinsic factors which can be represented by animal models, acquired characteristics are best explored through epidemiological studies. Levy [5] noted that though epidemiology captures many issues germane to CRA, there is presently little guidance available for its use in assessing cumulative risks. The NRC [6] however has highlighted the utility of social epidemiology to inform CRA while Levy [5] provides a conceptual framework for incorporating epidemiology into the process. In a recent paper, Schwartz *et al*. [48] discuss three challenges to studying differential vulnerability and susceptibility using epidemiological data and potential methods to address them. These challenges include disentangling complex interactions and synergies, incorporating spatially and temporally nested data, and quantifying risk inequalities to determine pockets of vulnerabilities.

To incorporate susceptibility into CRA, more and better data is needed to identify, characterize, and prioritize potential factors. Our analysis used SES as a potential effect modifier of lead toxicity because of its wide use in the epidemiological literature and its omnibus nature. SES serves as a proxy for numerous other potential lead-related susceptibility factors such as poor iron or calcium intake [49,50,51,52], high stress [53,54,55,56,57,58], cumulative exposures [59,60,61,62], or the presence of comorbidities [63]. From a policy perspective, SES as a variable is valuable for its potential to capture the adverse effects of multiple susceptibilities. The NRC recommends a focus on SES for this very reason [6]. We note though that concentrating on SES is less helpful for delineating specific pathways of effect and determining which are most important for modifying lead toxicity. Measuring susceptibility through SES may be better suited to environmental regulatory decisions such as setting a NAAQS; however public health programs and interventions implemented at a smaller scale would benefit from more focused and specific susceptibility measures.

Finally we note that even when data on susceptibility is too uncertain to realistically be used to set policy, examining the information quantitatively can still be a valuable endeavor. For example, although EPA performed a risk assessment to support the development of the lead NAAQS, uncertainties regarding the design of the assessment, estimation of lead concentrations in environmental media and blood, and calculations of lead-related IQ loss led the Agency to focus on an evidence-based approach rather than the derived risk estimates in making their final decisions. However, risk assessment results were still evaluated to provide additional perspective. As long as limits are known, imperfect data can still be considered in a decision context even if its value is questioned from a scientific point-of-view. The act of gathering and assembling data may itself be beneficial as the process can help to direct research efforts towards the types of data that will be most useful to policymakers for future decision-making.

## 5. Conclusions

In contrast to traditional risk assessment, CRA asks broader questions, is situated within a larger public health context, and addresses multiple health determinants simultaneously. As the methodology improves, CRA may become an increasingly valuable tool for policymakers, public health practitioners, and the public at-large. However CRA’s complexity is currently its greatest liability; the approach requires a strong research foundation across multiple disciplines. Without adequate data, incorporation of susceptibility into environmental policy remains a challenge. 

In conclusion, we found that the use of data developed in susceptible populations would result in the selection of a more protective lead NAAQS under the same decision framework applied by EPA. Our study raises important questions about the adequacy of current environmental standards for protecting all populations and underscores the need to develop guidance for incorporating susceptibility into public health research and policy. 
